# Natural history of retinal degeneration in ovine models of CLN5 and CLN6 neuronal ceroid lipofuscinoses

**DOI:** 10.1038/s41598-022-07612-7

**Published:** 2022-03-07

**Authors:** S. J. Murray, N. L. Mitchell

**Affiliations:** Faculty of Agriculture and Life Sciences, Lincoln University, PO Box 85084, Lincoln, Canterbury 7647 New Zealand

**Keywords:** Neurodegeneration, Retinal diseases

## Abstract

Neuronal ceroid lipofuscinoses (NCL; Batten disease) are a group of inherited neurodegenerative diseases with a common set of symptoms including cognitive and motor decline and vision loss. Naturally occurring sheep models of CLN5 and CLN6 disease display the key clinical features of NCL, including a progressive loss of vision. We assessed retinal histology, astrogliosis, and lysosomal storage accumulation in CLN5 affected (CLN5^−/−^) and CLN6 affected (CLN6^−/−^) sheep eyes and age-matched controls at 3, 6, 12, and 18 months of age to determine the onset and progression of retinal pathology in NCL sheep. The retina of CLN5^−/−^ sheep shows progressive atrophy of the outer retinal layers, widespread gliosis, and accumulation of lysosomal storage in retinal ganglion cells late in disease. In contrast, CLN6^−/−^ retina shows significant atrophy of all retinal layers, progressive gliosis, and earlier accumulation of lysosomal storage. This study has highlighted the differential vulnerability of retinal layers and the time course of retinal atrophy in two distinct models of NCL disease. This data will be valuable in determining potential targets for ocular therapies and the optimal timing of these therapies for protection from retinal dysfunction and degeneration in NCL.

## Introduction

Neuronal ceroid lipofuscinoses (NCL; Batten disease) are a group of recessively inherited lysosomal storage disorders. NCL presents primarily as a progressive neurodegenerative disease in children and is ultimately fatal. There are currently 13 known variants of NCL, designated CLN1-8, and CLN10-14. Common symptoms across most variants of NCL include cognitive and motor regression, seizures, and progressive loss of vision.

Vision loss in NCL is thought to be due to cell death in both the visual cortex and in the retina. Cortical atrophy and neuronal cell loss is most pronounced in the occipital lobe of patients at end-stage disease^1^. The diseased retina has been observed to be no more than a glial scar at post-mortem, however the onset and progression of retinal cell loss in human patients is still unclear^1^. Several animal models of NCL, including rodents, canines, and livestock demonstrate similar visual deficits and retinal pathology to NCL patients, making them valuable tools in studying the retinal component of disease^2–7^. Naturally occurring models of NCL exist in sheep in New Zealand; CLN5 disease in Borderdale sheep, and CLN6 disease in South Hampshire sheep^8,9^. These models replicate the primary symptomatic and neurological profiles of NCL, including a progressive loss of vision^10–12^.

Many of the studies of retinal tissue from NCL patients or models have been done at end stage disease, therefore a detailed analysis of the onset and progression of retinal dystrophy in NCL is still lacking. Understanding the differential vulnerability of retinal cells at distinct disease stages will be useful in determining the main target cells and optimal timing of potential therapeutics. This comprehensive natural history study assesses retinal histology and lysosomal storage burden in two different sheep models of NCL between pre-symptomatic and end-stage disease.

## Results

### Sheep eye dimensions in health and disease

**Figure 1:**
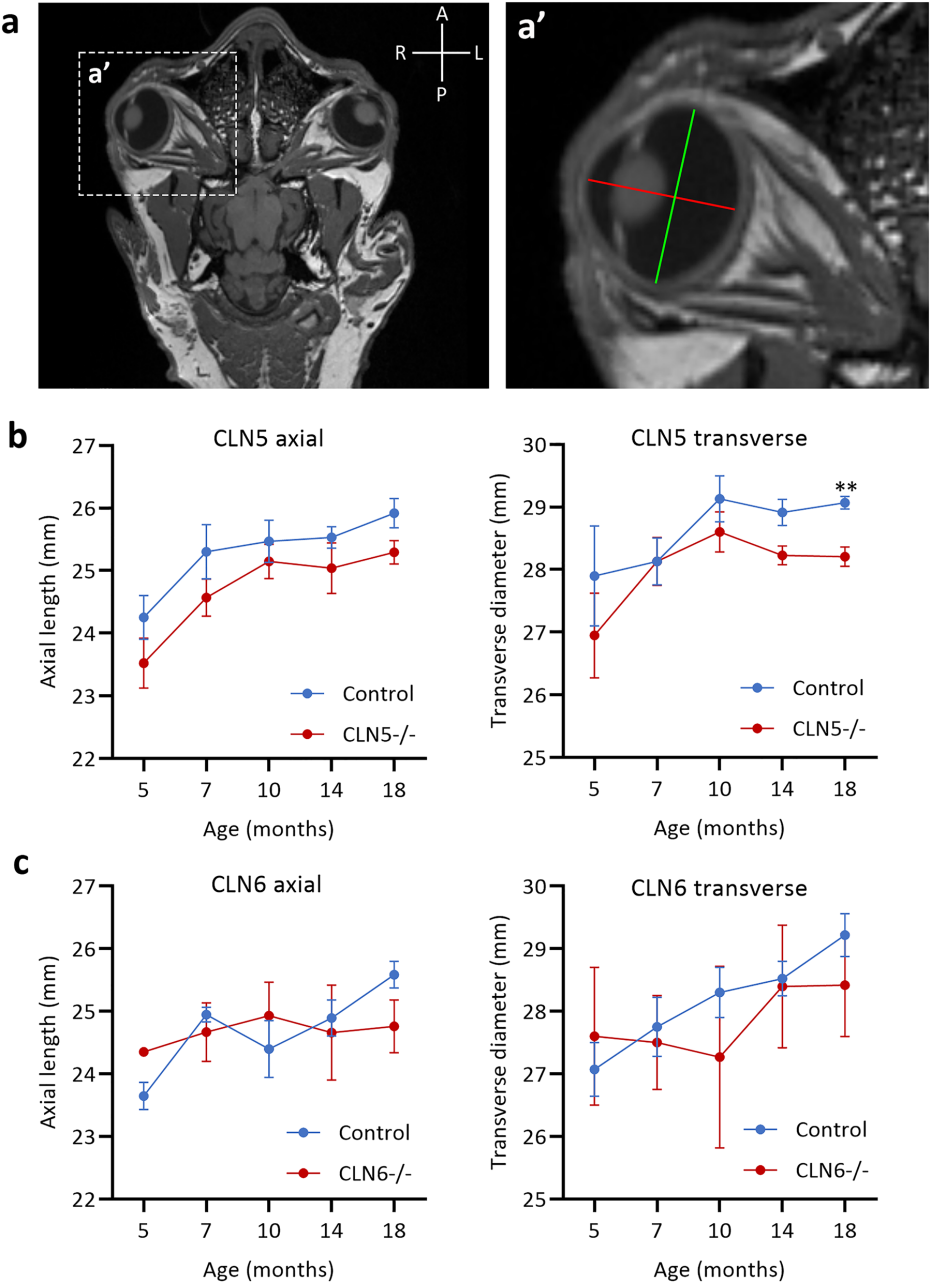
Eye globe dimensions in healthy and NCL affected sheep. (**a**) Axial MR image of sheep head at the level of the eyes. Dashed box indicates zoomed image (**a’**). (**a’**) Axial MR image of the eye globe indicating how measurements for axial length (mm; red line) and transverse diameter (mm; green line) were obtained. (**b**) Mean ± SEM eye axial length and transverse diameter measurements in healthy (Control, n = 3, blue) and CLN5 affected (CLN5^−/−^, n = 4, red) Borderdale sheep. **indicates* p* < 0.001. (**c**) Mean ± SEM eye axial length and transverse diameter measurements in healthy (Control, n = 4, blue) and CLN6 affected (CLN6^−/−^, n = 3, red) South Hampshire sheep.

Measurements of the sheep eye globe were obtained from axial MRI scans of the sheep head between 5 and 18 months of age (Fig. 1a). In healthy Borderdale control sheep the axial length of the eye was 24.3 ± 0.35 mm at 5 months of age and grew to 25.9 ± 0.24 mm by 18 months of age, with the CLN5^−/−^ sheep eye following a similar trajectory. The transverse diameter of the healthy sheep eye was 27.9 ± 0.8 mm at 5 months of age and had grown to 29.1 ± 0.1 mm by 18 months of age. In the CLN5^−/−^ sheep eye the transverse diameter was similar to healthy sheep between 5 and 10 months of age, then began to diverge and was significantly lower (28.2 ± 0.16 mm) than healthy sheep at 18 months of age (Fig. 1b). There was no significant difference in axial length or transverse diameter in the healthy South Hampshire control sheep compared to the CLN6^−/−^ sheep eye between 5 and 18 months of age (Fig. 1c). Across both breeds, the average axial length of the healthy adult (18 m) sheep eye was 25.7 ± 0.16 mm while the average transverse diameter of the adult sheep eye was 29.1 ± 0.19 mm.

### Retinal pathology in sheep with CLN5 disease

**Figure 2:**
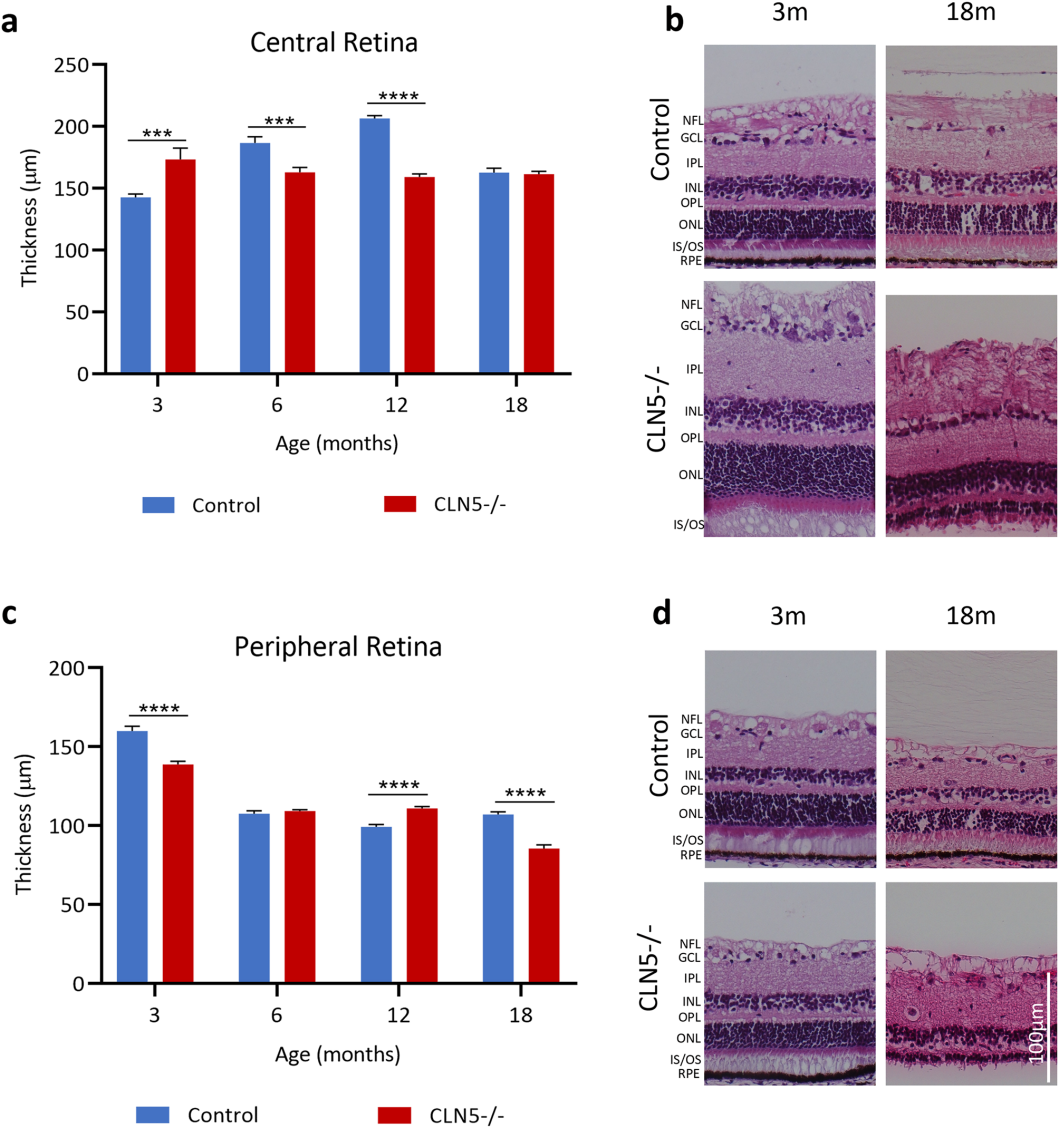
Total retinal thickness in healthy control and CLN5^−/−^ sheep at different ages. (**a**) Mean ± SEM total thickness (µm) of the central retina at 3, 6, 12, and 18 months of age in control (n = 2–4, blue) and CLN5^−/−^ (n = 2–4, red) sheep. (**b**) Representative images of Hematoxylin and Eosin (H + E) stained central retina in control and CLN5^−/−^ sheep at 3 and 18 months of age. (**c**) Mean ± SEM total thickness (µm) of the peripheral retina at 3, 6, 12, and 18 months of age in control (n = 2–4, blue) and CLN5^−/−^ (n = 2–4, red) sheep. (**d**) Representative images of H + E stained peripheral retina in control and CLN5^−/−^ sheep at 3 and 18 months of age. ***p < 0.001, ****p < 0.0001. NFL; nerve fibre layer, GCL; ganglion cell layer, IPL; inner plexiform layer, INL; inner nuclear layer, OPL; outer plexiform layer, ONL; outer nuclear layer, IS/OS; inner and outer segments of photoreceptors, RPE; retinal pigment epithelium. Scale bar 100 µm.

Total retinal thickness was analysed in both the central and peripheral retina of control and CLN5^−/−^ sheep. In control animals, the thickness of central retina grows from approximately 142 ± 2.6 µm at 3 months of age and peaks at 206 ± 2.2 µm at 12 months of age. Conversely, the retina of CLN5^−/−^ sheep is highest at 3 months of age (173 ± 9.1 µm) and declines steadily to 161 ± 2.2 µm at 18 months of age (Fig. 2a, b). The thickness of the peripheral retina of both control and CLN5^−/−^ sheep declines with age, although at 18 months of age the peripheral retina in control sheep (107 ± 1.7 µm) is significantly thicker compared to CLN5^−/−^ sheep (85.4 ± 2.3 µm; Fig. 2c, d).

**Figure 3:**
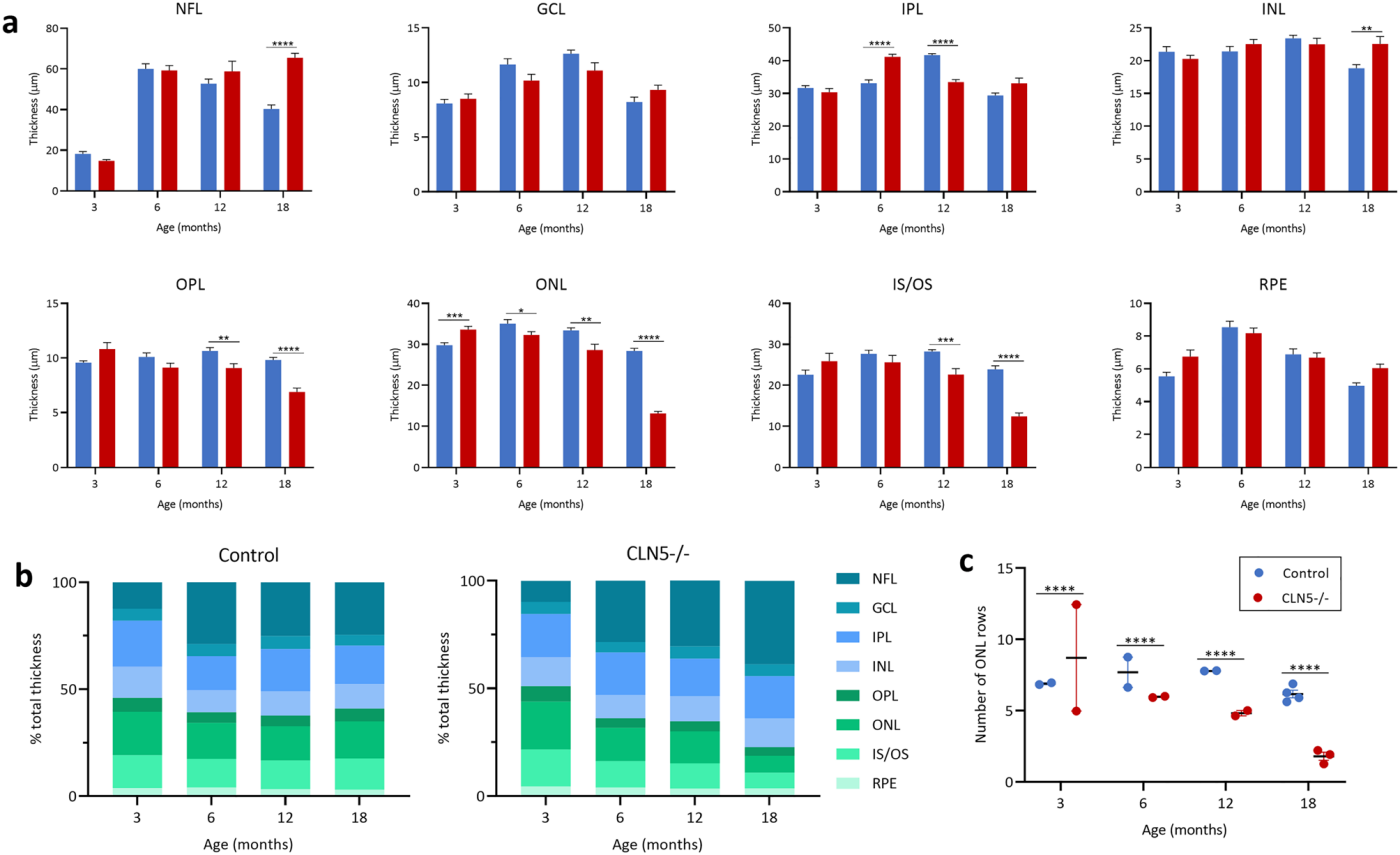
Thickness of individual retinal layers in healthy control and CLN5^−/−^ sheep at different ages. (**a**) Mean ± SEM thickness of individual layers in the central retina between control (n = 2–4, blue) and CLN5^−/−^ (n = 2–4, red) sheep at 3, 6, 12 and 18 months of age. (**b**) Percentage of total retinal thickness represented by each layer in control and CLN5^−/−^ sheep at various ages. (**c**) Mean ± SEM number of rows of cells in the outer nuclear layer (ONL) in control and CLN5^−/−^ sheep at various ages. *p < 0.05, **p < 0.01, ***p < 0.001, ****p < 0.0001. NFL; nerve fibre layer, GCL; ganglion cell layer, IPL; inner plexiform layer, INL; inner nuclear layer, OPL; outer plexiform layer, IS/OS; inner and outer segments of photoreceptors, RPE; retinal pigment epithelium.

Although the total central retinal thickness is comparable between control and CLN5^−/−^ sheep at 18 months of age, representative images (Fig. 2b) and individual layer measurements (Fig. 3) show that there is a significant reduction in the outer plexiform and nuclear layers (OPL/ONL) and inner and outer photoreceptor segments (IS/OS), but a significantly enlarged nerve fibre layer (NFL) in CLN5^−/−^ sheep compared to controls. In comparison to the outer retina, there is only minor differences in inner plexiform and nuclear layer thicknesses between control and CLN5^−/−^ sheep at each age (Fig. 3). At 3 months of age the proportion of total retinal thickness taken up by each retinal layer is comparable between control and CLN5^−/−^ sheep, however, by 18 months of age CLN5^−/−^ sheep have a higher proportion of NFL (38%) compared to controls (24%), and lower proportions of ONL (7%) and IS/OS (7%) compared to controls (17% and 14% respectively; Fig. 3b).

The number of rows of nuclei in the ONL was quantified in the central retina of control and CLN5^−/−^ sheep at each age (Fig. 3c). One CLN5^−/−^ sheep analysed had a very high number of rows (12.5 ± 0.17) at 3 months of age, leading to a significantly higher average number of rows in CLN5^−/−^ sheep compared to controls at this age. However, at 6, 12, and 18 months the number of ONL rows was significantly lower in CLN5^−/−^ sheep compared to controls, with ONL rows remaining stable at 6.9 ± 0.05 rows in controls over time but declining to an average of 1.7 ± 0.06 rows in CLN5^−/−^ sheep by 18 months of age.

**Figure 4:**
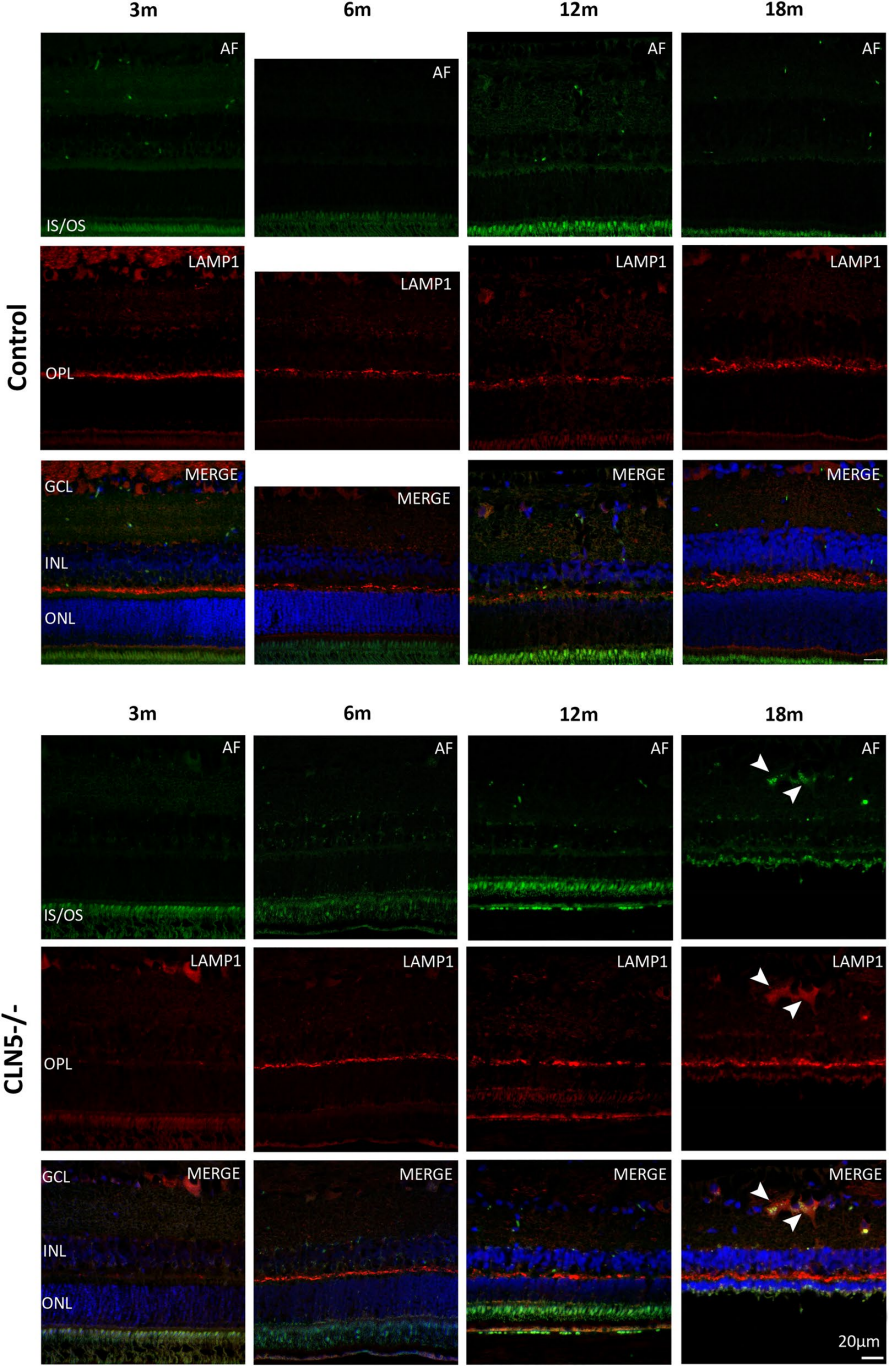
Lysosomal storage burden in the retina of healthy control and CLN5^−/−^ sheep at different ages. Top panel; Representative confocal images of control central retina. Bottom panel; Representative confocal images of CLN5^−/−^ central retina. AF; autofluorescence (green). LAMP1; lysosomal-associated membrane protein 1 (red). Merged image shows AF and LAMP1 with DAPI nuclei stain (blue). Arrowheads highlight co-localisation of AF and LAMP1, indicating lysosomal storage in the CLN5^−/−^ retina at 18 m (m; months of age). GCL; ganglion cell layer, INL; inner nuclear layer, OPL; outer plexiform layer, ONL; outer nuclear layer, IS/OS; inner and outer segments of photoreceptors. Scale bar 20 µm.

Autofluorescent (AF) signal in the central retina was studied in conjunction with lysosomal protein LAMP1 to determine the extent of lysosomal storage burden in the CLN5^−/−^ retina compared to controls (Fig. 4). In the control retina AF was minimal at all ages, with signal coming primarily from the IS/OS, while LAMP1 was concentrated to the ganglion cell layer (GCL), OPL, and retinal pigment epithelium (RPE). In CLN5^−/−^ retina there was minimal AF signal at 3 and 6 months of age, while at 12 months of age some AF puncta were evident in the IPL and OPL but were not co-localised with LAMP1 (Fig. 4). By 18 months of age AF signal was evident throughout the CLN5^−/−^ retina, particularly in the GCL where it was co-localised with LAMP1 in ganglion cell bodies (Fig. 4, arrowheads).

Retinal gliosis was assessed by GFAP staining in the central retina. In control animals, resident astrocytes with horizontally elongated cell bodies and processes were evident in the NFL at all ages but absent from all other layers (see Supplementary Fig. S1). GFAP-positive astrocytes were also present in the NFL of CLN5^−/−^ animals however they were morphologically irregular in shape and their processes had a more disorganised appearance compared to the control NFL. From 6 months of age, GFAP immunoreactivity had spread to the outer retinal layers of the diseased eye, in the form of vertically elongated Müller cell processes, with some Müller cell bodies evident by 18 months of age (see Supplementary Fig. S1) suggesting heightened inflammation.

### Retinal pathology in sheep with CLN6 disease

**Figure 5:**
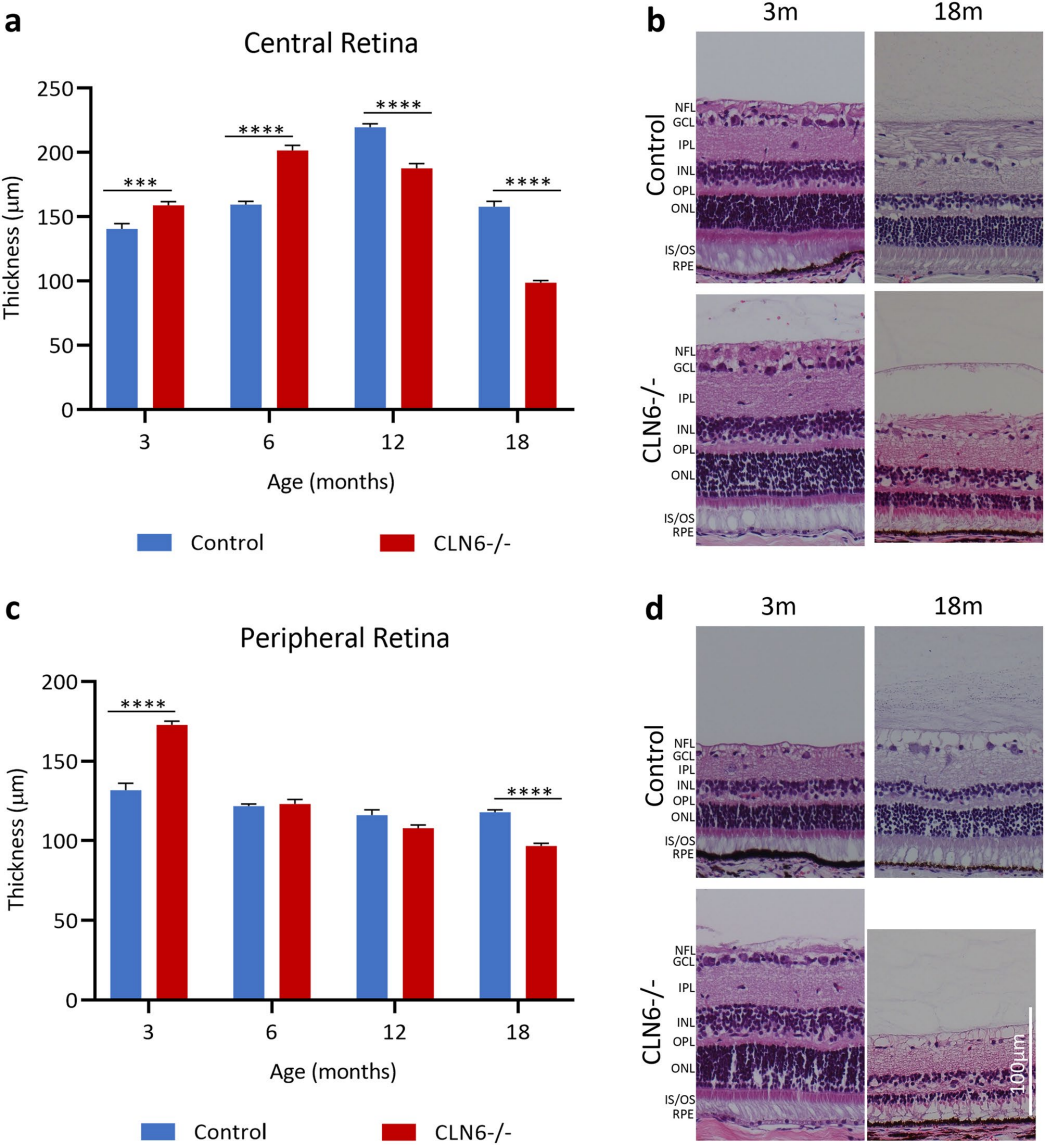
Total retinal thickness in healthy control and CLN6^−/−^ sheep at different ages. (**a**) Mean ± SEM total thickness (µm) of the central retina at 3, 6, 12, and 18 months of age in control (n = 2–4, blue) and CLN6^−/−^ (n = 2–5, red) sheep. (**b**) Representative images of Hematoxylin and Eosin (H + E) stained central retina in control and CLN6^−/−^ sheep at 3 and 18 months of age. (**c**) Mean ± SEM total thickness (µm) of the peripheral retina at 3, 6, 12, and 18 months of age in control (n = 2–4, blue) and CLN6^−/−^ (n = 2–5, red) sheep. (**d**) Representative images of H + E stained peripheral retina in control and CLN6^−/−^ sheep at 3 and 18 months of age. ***p < 0.001, ****p < 0.0001. NFL; nerve fibre layer, GCL; ganglion cell layer, IPL; inner plexiform layer, INL; inner nuclear layer, OPL; outer plexiform layer, ONL; outer nuclear layer, IS/OS; inner and outer segments of photoreceptors, RPE; retinal pigment epithelium. Scale bar 100 µm.

Total retinal thickness was analysed in both the central and peripheral retina of control and CLN6^−/−^ sheep. In control sheep, the central retina grew initially to reach a peak thickness of 219 ± 2.6 µm at 12 months of age. The central retina of CLN6^−/−^ sheep also grew but reached a peak of 201 ± 3.9 µm at 6 months of age (Fig. 5a, b). Peripheral retina of control sheep remained relatively stable between 3 and 18 months of age. Conversely, CLN6^−/−^ sheep had significantly thicker peripheral retina at 3 months of age, showed a sharp decline between 3 and 6 months of age, and was significantly thinner (96 ± 1.6 µm) than control (117 ± 1.5 µm) peripheral retina at 18 months of age (Fig. 5c, d).

**Figure 6:**
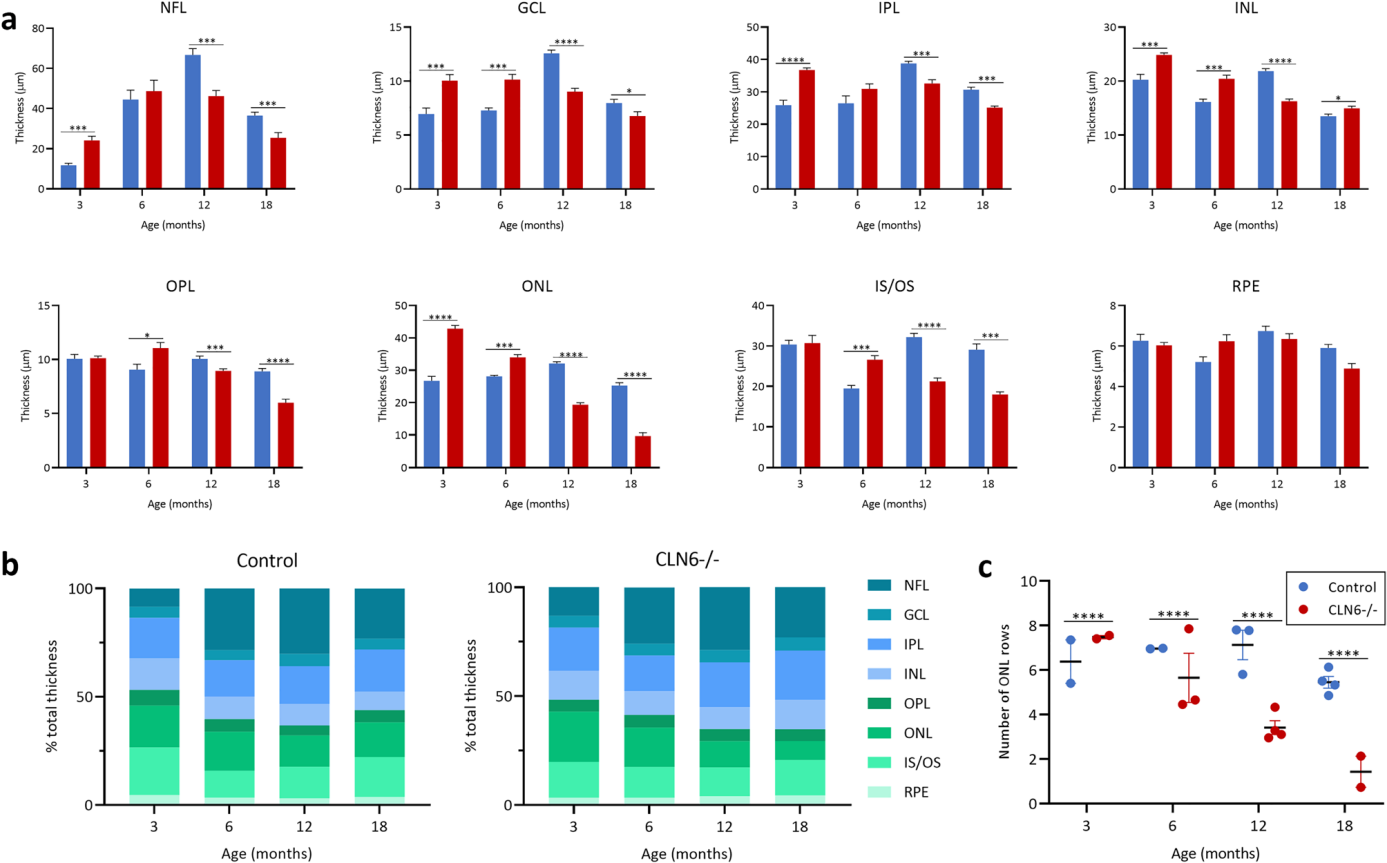
Thickness of individual retinal layers in healthy control and CLN6^−/−^ sheep at different ages. (**a**) Mean ± SEM thickness of individual layers in the central retina between control (n = 2–4, blue) and CLN6^−/−^ (n = 2–5, red) sheep at 3, 6, 12 and 18 months of age. (**b**) Percentage of total retinal thickness represented by each layer in control and CLN6^−/−^ sheep at various ages. (**c**) Mean ± SEM number of rows of cells in the outer nuclear layer (ONL) in control and CLN6^−/−^ sheep at various ages. *p < 0.05, ***p < 0.001, ****p < 0.0001. NFL; nerve fibre layer, GCL; ganglion cell layer, IPL; inner plexiform layer, INL; inner nuclear layer, OPL; outer plexiform layer, IS/OS; inner and outer segments of photoreceptors, RPE; retinal pigment epithelium.

Looking at individual layers of the central retina, inner retinal layers (NFL, GCL, IPL and INL) were significantly thicker in CLN6^−/−^ animals compared to controls early in disease, but were significantly thinner by end stage disease at 18 months of age (Fig. 6a). A similar pattern was observed in the outer retinal layers, with the exception of the RPE which remained relatively unchanged between 3 and 18 months. The proportion of total retinal thickness taken up by each retinal layer was comparable between control and CLN6^−/−^ sheep at 3 and 18 months of age with the only notable difference being the ONL, which makes up 16% of total thickness in control sheep and only 8% of total thickness in CLN6^−/−^ sheep at 18 months of age (Fig. 6b). The number of ONL rows was significantly lower in CLN6^−/−^ sheep compared to controls from 6 months of age onwards, with a diseased average of only 1.4 ± 0.08 rows evident by 18 months of age (Fig. 6c).

**Figure 7:**
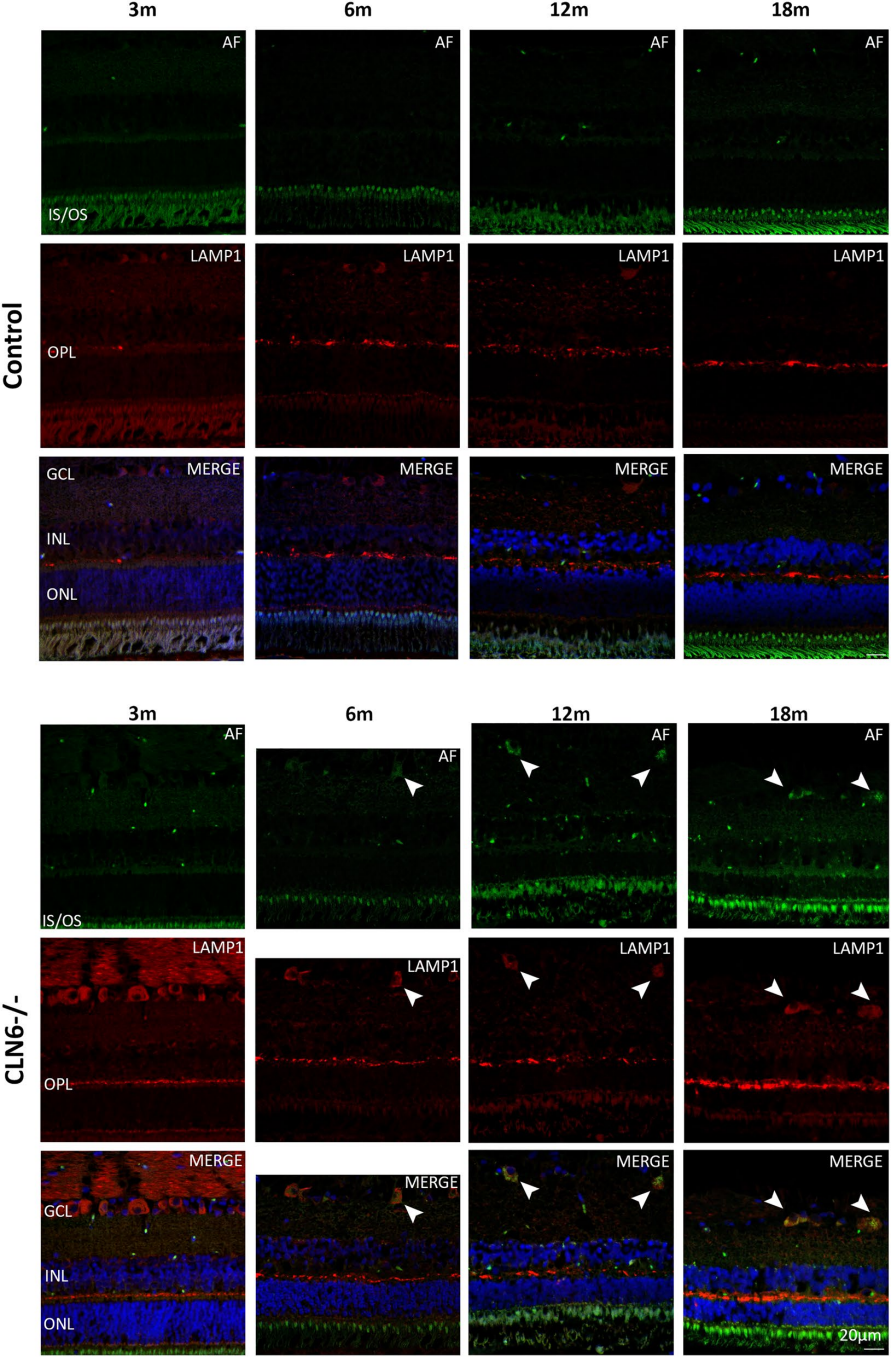
Lysosomal storage burden in the retina of healthy control and CLN6^−/−^ sheep at different ages. Top panel; Representative confocal images of control central retina. Bottom panel; Representative confocal images of CLN6^−/−^ central retina. AF; autofluorescence (green). LAMP1; lysosomal-associated membrane protein 1 (red). Merged image shows AF and LAMP1 with DAPI nuclei stain (blue). Arrowheads highlight co-localisation of AF and LAMP1, indicating lysosomal storage in the CLN6^−/−^ retina at 12 and 18 months of age. GCL; ganglion cell layer, INL; inner nuclear layer, OPL; outer plexiform layer, ONL; outer nuclear layer, IS/OS; inner and outer segments of photoreceptors. Scale bar 20 µm.

Accumulation of AF signal was minimal in control central retina across all ages. Sparse AF puncta were observed in the control retina at 12 and 18 months of age however these did not co-localise with LAMP1. In the CLN6^−/−^ retina, AF signal was evident from 3 months of age but did not co-localise with LAMP1 at this age. By 6 months of age there was faint AF signal in LAMP1-positive ganglion cell bodies in the CLN6^−/−^ retina, which became stronger and more distinct at 12 and 18 months of age (Fig. 7, arrowheads).

GFAP immunoreactivity was confined to the quiescent elongated astrocytes of the NFL in the central retina of control sheep and showed minor increases in intensity with age (see Supplementary Fig. S2). Whilst GFAP signal was also initially localised to the same NFL cells in the CLN6^−/−^ retina, these cells had irregularly shaped soma and thickened processes. Vertically oriented Müller cell processes were observed in the outer retina of CLN6^−/−^ animals from 6 months of age, with Müller cell bodies evident from 12 months of age (see Supplementary Fig. S2).

## Discussion

This study details the progression of retinal degeneration from pre-symptomatic to end-stage disease in two distinct sheep models of NCL; CLN5 disease in Borderdale sheep and CLN6 disease in South Hampshire sheep. Total retinal thickness, individual layer thickness, gliosis, and accumulation of autofluorescent storage material were assessed in post-mortem eyes from diseased sheep and age-matched healthy controls. Four ages (3, 6, 12 and 18 months) were selected to represent four distinct clinical disease stages, being pre-symptomatic, early symptomatic, symptomatic and end-stage disease respectively.

Total retinal thickness was assessed in both the central and peripheral retina, with the central retina showing an earlier and more distinct difference between control and affected sheep. In both breeds the central retina was thicker in affected animals compared to controls at 3 months of age. This may be due to early compensatory mechanisms or inflammatory edema in the retina of affected animals, however requires more investigation. Compensatory mechanisms may include upregulated mRNA or protein expression of neuroprotective markers and could be assessed by molecular or histological analysis of pre-symptomatic sheep retina. Although early increases in retinal thickness have not been observed in other animal models of NCL, they have been observed in several degenerative diseases including age-related macular degeneration, optic neuritis, diabetic retinopathy, and leber congenital amaurosis^13–16^. In many of these diseases the early increase in retinal thickness has been attributed to inflammatory edema which occurs in response to photoreceptor cell stress prior to retinal atrophy^13–16^. Although early upregulation of GFAP was not observed in affected sheep retina, it would be useful in future to assess these retina for other signs of inflammation such as microglial infiltration, or upregulation of Müller cell specific markers or inflammatory cytokines.

There was early growth of the central retina in control Borderdale sheep leading to thicker retina in control compared to CLN5^−/−^ sheep from 6 months onwards, however growth was slower in control South Hampshire sheep. Despite initial differences in peripheral retina thickness between control and affected sheep of both breeds, the thicknesses were similar from 6 months of age until a significant drop in thickness in CLN5^−/−^ and CLN6^−/−^ sheep at end-stage disease. While total retinal thickness is informative for tracking global changes in the retina, it is important to consider the structure and function of individual layers of the retina and how these change over the course of disease. This is particularly pertinent in CLN5^−/−^ retina at end stage disease where total thickness is comparable to controls, however almost 40% of this total thickness is taken up by the NFL. Meanwhile there is severe shrinkage of the ONL and IS/OS, which is more likely to be the underlying cause of retinal dysfunction. The cause and physiological consequences of this increasing NFL thickness in affected animals is unknown. In CLN5^−/−^ sheep it is primarily the outer retinal layers (OPL, ONL, and IS/OS) which show significant degeneration over the disease course. While inner retina (GCL, IPL, INL) thicknesses do fluctuate over time, they are more comparable to control at end stage disease. In contrast, all retinal layers in CLN6^−/−^ sheep significantly degenerate over the course of the disease, except for the RPE.

In both breeds, lysosomal storage occurs primarily in ganglion cell bodies, however it appears earlier in the retina of CLN6^−/−^ sheep compared to CLN5^−/−^ sheep. Minor amounts are observed in the CLN6^−/−^ retina at 6 months of age, which then progresses until ganglion cell bodies appear full of storage by end stage disease. This is in keeping with early studies of the CLN6^−/−^ sheep retina, which showed atrophy of photoreceptor cell bodies and inner and outer segments, and accumulation of autofluorescent storage material primarily in ganglion cells^10,11^.

In the control sheep retina at all ages studied, GFAP immunoreactivity was primarily confined to horizontally elongated cell bodies and processes in the NFL, reminiscent of quiescent astrocytes commonly found in the healthy retina^17,18^. However, in affected sheep NFL resident astrocytes had an altered morphology and, from 6 months of age, GFAP-positive cells were evident throughout the retina. Müller cells typically do not express GFAP, but upregulate its expression in response to pathological processes in the retina^19^. GFAP-expressing Müller cells were evident in the outer retina of affected animals from 6 months of age, indicating the initiation of reactive gliosis. Early and progressive increases in GFAP immunoreactivity and activation of Müller cells has also been observed in the retina of several mouse models of NCLs^20–23^.

A significant reduction in ERG a- and b-waves in CLN5 and CLN6 affected sheep occurs from 9 to 11 months of age, suggesting dysfunction of photoreceptor and bipolar cells^12^. This coincides with the age when clinical signs of visual deterioration, such as reduced menace response and pupillary light reflex, also begin to occur in affected sheep^12,24^. By 15 to 17 months of age ERG responses are abolished in the majority of affected sheep and they can no longer navigate through a maze^12,24^. The retinal pathology presented in this study aligns with these clinical features as the number of ONL rows, indicative of photoreceptor cell body numbers, show significant decline from 6 months of age and diminish over the disease course.

Post-mortem data from retina tissue of NCL patients is rare, however what is known about the retina of CLN5 and CLN6 patients is summarised in Table 1. The results presented here from CLN6 sheep align with what is observed in CLN6 patients as all retinal layers in affected sheep show progressive atrophy and accumulation of lysosomal storage in ganglion cells. In CLN5 sheep, no ganglion cell loss was observed as it is in human CLN5, however accumulation of lysosomal storage and outer retinal atrophy in sheep aligns with pathology in CLN5 patients. Visual impairment often presents in early to mid-disease in CLN5 and classical CLN6 patients, with abolished ERGs reported between 7 and 10 years of age in CLN5, and visual failure between 3 and 6 years in CLN6^25,26^.

**Table 1:** Comparison of retinal disease in CLN5 and CLN6 sheep and human patients.

Retinal layer	CLN5^−/−^ sheep	CLN6^−/−^ sheep	Human CLN5*	Human CLN6*	References
NFL	No atrophy, thicker than control at 18 m	Atrophy from 12 m onwards			
GCL	No atrophy, storage at 18 m	Thicker initially, atrophy from 12 m, storage increasing from 6 m	Significant atrophy, high storage in surviving cells	High levels of storage	^1,27^
IPL	Atrophy at 12 m but does not worsen	Thicker initially, atrophy from 12 m			
INL	No atrophy, thicker than control at 18 m	Variable—thicker at 18 m	Moderate atrophy	Extensive cell loss	^1,27^
OPL	Atrophy from 12 m onwards	Atrophy from 12 m onwards			
ONL	Atrophy from 6 m onwards—severe at 18 m	Thicker initially, atrophy from 12 m onwards	Moderate atrophy	Extensive cell loss	^1,27^
IS/OS	Atrophy from 12 m onwards	Thicker initially, atrophy from 12 m onwards		Extensive cell loss	^27^
RPE	No atrophy	No atrophy			
**Clinical manifestation**	Declining ERG from 9 to 11 m, reduced visual clinical score from 10 m	Declining ERG from 9 to 11 m, reduced visual clinical score from 7 m	Early visual impairment, ERG abolished by 7–10 y	Visual failure by 3–6 y, ERG abolished early	^12,25–27^

*Data refers to cases of late infantile onset NCL.

ERG; electroretinography, m; months, y; years, NFL; nerve fibre layer, GCL; ganglion cell layer, IPL; inner plexiform layer, INL; inner nuclear layer, OPL; outer plexiform layer, ONL; outer nuclear layer, IS/OS; inner and outer segments of photoreceptors, RPE; retinal pigment epithelium.

The sheep eye is increasingly being recognised as a good model for studying human retinal disease and for testing ocular therapies^28–31^, due to its similarities in globe size and retinal structure to humans. There are several different potential approaches for delivering corrective therapies to the retina in NCL, including intravitreal, subretinal, suprachoroidal, periocular, or topical administration. Of these, intravitreal delivery is one of the least invasive as it involves injection of the drug to the vitreous cavity of the eye, but has less target specificity than a subretinal or suprachoroidal delivery route. While the intravitreal route is not specifically targeted to the outer retina and has barriers such as the inner limiting membrane (ILM) to overcome there are several reasons to consider this approach for retinal diseases caused by soluble protein deficiencies. For example, in CLN5 disease if only inner retinal cells were corrected through intravitreal therapy they could secrete functional CLN5 protein to other retinal cells, a mechanism known as cross-correction^32^. In addition, given the fragility of the diseased retina it is likely the ILM is disrupted and more permeable in diseased eyes, and in fact others have shown better viral vector transduction in diseased eyes compared to wild-type^33^. Intravitreal drug delivery is commonplace in clinical settings for a range of ocular diseases^34–36^, and retinal dysfunction and pathology was successfully ameliorated up to 18 months of age following a single intravitreal injection of CLN5 gene therapy in CLN5^−/−^ sheep^37^. Biotechnology company Neurogene Inc has recently announced the planned initiation of a Phase I/II clinical trial for CLN5 disease utilising both intracerebroventricular and intravitreal delivery routes^38^ and a clinical trial is underway for the intravitreal delivery of the CLN2 enzyme replacement therapy Brineura (ClinicalTrials.gov identifier: NCT05152914).

The outlook for treating the retinal component of CLN6 disease is more complicated as we have shown that all layers of the retina significantly degenerate in CLN6 affected sheep, and CLN6 is a membrane bound protein meaning cross-correction is unlikely to be of benefit. Indeed, a recent trial of intravitreal gene therapy in CLN6^−/−^ sheep showed some attenuation of pathology but no rescue of visual function^37^. A combination of approaches such as suprachoroidal and intravitreal may be appropriate in this case as suprachoroidal would target outer retinal cells with a lower risk of retinal detachment, and the intravitreal route would target inner retinal cells. The general consensus on timing of treatment for degenerative diseases is often ‘the earlier the better’. Our results in NCL sheep have reaffirmed this, as atrophy of retinal layers and accumulation of lysosomal storage start becoming evident by 6 months of age. In addition, early treatment is preferable if using the intravitreal approach as the ILM is still developing and therefore more permeable in younger animals^39,40^.

## Conclusions

This systematic study of the progression of retinal degeneration in distinct sheep models of CLN5 and CLN6 NCL has highlighted the differential vulnerability of retinal cell types in each disease and the time course of degeneration. In CLN5 sheep, the outer retina is most severely affected, with significant thinning of the outer nuclear layer evident from 6 months of age. Conversely in CLN6 sheep, all retinal layers show significant degeneration from 12 months of age. Lysosomal storage accumulation in retinal ganglion cells was a common feature in both sheep models, which is in keeping with observations of post-mortem human retina. This histological data, in addition to assessment of sheep eye globe dimensions, further validates the use of sheep to study the retinal component of NCL and potential ocular therapies to attenuate retinal dysfunction and degeneration.

## Methods

### Animals

Borderdale and South Hampshire sheep were diagnosed at birth^8,9^ and maintained at the Lincoln University research farm under US National Institutes of Health guidelines for the care and use of animals in research and the NZ Animal Welfare Act (1999). Homozygous affected ewes from both breeds (CLN5^−/−^, n = 2–4, CLN6^−/−^, n = 2–5) were sacrificed at 3, 6, 12, and 18 months of age along with age-matched healthy heterozygous flockmates as controls (CLN5^+/−^ n = 2–4, CLN6^+/−^ n = 2–4). All experimental protocols were approved by the Lincoln University Animal Ethics Committee. All studies are reported in accordance with the ARRIVE Essential 10 guidelines.

### Tissue processing

Sheep were sacrificed by penetrating captive bolt to the cervical spine followed by rapid exsanguination. Eye globes were enucleated at the time of death, fixed in 10% formalin for a minimum of 2 h, and sent to Gribbles Veterinary Pathology, Christchurch, NZ for post-fixation in Bouin’s solution (Sigma-Aldrich, St Louis, MO, USA, HT10132) for 4 h, followed by wax embedding. Retinal paraffin sections were cut at 3 µm, mounted and a subset stained with Hematoxylin and Eosin (H + E) histological stain by Gribbles Veterinary Pathology for analysis of retinal thickness and layer differentiation.

### Immunohistochemistry

Retinal paraffin sections were stained with either lysosome-associated membrane protein 1 (LAMP1; 1:500; Abcam ab24170, Cambridge, UK) or glial fibrillary acidic protein (GFAP; 1:2500, Dako Z0334, Glostrup, Denmark). Sections were de-waxed twice in xylene (5 min) and rehydrated through a graded ethanol series to water. Following re-hydration, sections underwent antigen retrieval in 10 mM Sodium Citrate buffer (pH 6) at 80 °C for 30 min, then were allowed to cool for 20 min. Sections were then washed in TBST (Tris-buffered saline, pH 7.6, containing 0.3% Triton X-100) and blocked in 10% Normal Goat Serum (NGS) in TBST for 30 min at room temperature, before overnight incubation in the primary antibody in 10% NGS in TBST at 4 °C. Sections were washed in TBST and then incubated in goat anti-rabbit Alexa Fluor® 594 secondary antibody (1:500; Invitrogen A-11012, Carlsbad, CA, USA) 10% NGS in TBST, 1 h, in the dark at room temperature. Sections were then washed and incubated in DNA stain DAPI (4′,6-diamidino-2-phenylindole dihydrochloride; Sigma 10236276001) for 3 min at room temperature. DAPI was washed off in dH_2_0 and sections were coverslipped in BrightMount plus anti-fade mounting medium (Abcam ab103748).

### Imaging and analysis

All H + E stained sections were imaged on a Nikon Eclipse 50i microscope (Nikon Instruments Inc., Tokyo, Japan) paired to a Nikon Digital Sight DS-U3 camera and NIS-Elements BR software (v. 4.50 Nikon Instruments). Ten total retinal thickness measurements per eye were taken by a blinded researcher from the surface of the nerve fibre layer (NFL) to the base of the retinal pigment epithelium (RPE) in both the central retina (within 5 mm of the optic nerve head) and the peripheral retina (10–20 mm from the optic nerve head). Individual retinal layer thicknesses were calculated from ten measurements per eye taken from the central retina. Immunofluorescent GFAP signal was imaged using a CY3 Brightline 531 excitation/593 emission filter set (CY3-4040C; Semrock Inc. IDEX Corporation, IL, USA).

Co-localisation of LAMP1 and autofluorescent signal was assessed using immunofluorescent images captured on a Zeiss 510 laser scanning confocal microscope with Zen 2009 imaging software (Carl Zeiss Microscopy, Jena, Germany). Image collection parameters (laser power, scan speed, pixel dwell time, detector gain, and pinhole size) were optimised for each channel and remained consistent for all sections in each staining run. High magnification Z-stacks were captured using a 40x/NA 1.3 oil objective and post-processing was performed in ImageJ (National Institutes of Health, Bethesda, MD, USA; https://imagej.nih.gov/ij/).

### Eye globe measurements

Measurements of axial length and transverse diameter of the sheep eye globe were taken from an existing set of MRI images of healthy CLN5^+/−^ (n = 3), diseased CLN5^−/−^ (n = 4), healthy CLN6^+/−^ (n = 4) and diseased CLN6^−/−^ (n = 3) sheep obtained at 5, 7, 10, 14 and 18 months of age. Briefly, sheep were scanned in a 3-Tesla Skyra MRI scanner (Siemens, Erlangen, Germany), with a 20-channel head coil. Sheep were sedated by intravenous injection of 0.5 mg/kg live weight diazepam (Troy Laboratories NZ Pty Ltd, Auckland, NZ) and 10 mg/kg live weight ketamine (Phoenix Pharm Distributors Ltd, Auckland, NZ) prior to endotracheal intubation and maintenance on inhalation anaesthesia (isoflurane in oxygen, 1.5–3% v/v to effect). Sheep were placed in right lateral recumbency on the patient bed for scanning. DICOM files of T1-weighted scans were loaded into 3D Slicer (www.slicer.org, ^41^) and measurements were taken in the axial plane image using the ‘create line markup’ tool.

### Statistics

All statistical analysis was performed on GraphPad Prism© (v 8.2.0, GraphPad Software, San Diego, CA, USA, www.graphpad.com).

Results are reported as mean ± the standard error of the mean (SEM). Differences between healthy control and NCL affected sheep were assessed using two-tailed unpaired t-tests at each age (alpha = 0.05). Multiple comparisons were corrected for using the Holm-Sidak method. No statistical comparisons across ages were made as different animals were used at each age.

## Supplementary Information

Supplementary Information.
