# Supplementary material for: Natural history of retinal degeneration in ovine models of CLN5 and CLN6 neuronal ceroid lipofuscinoses
Source: Sci Rep. 2022 Mar 7;12:3670. doi: 10.1038/s41598-022-07612-7 (PMC8901734; doi:10.1038/s41598-022-07612-7)
Supplement: Supplementary file 1 — Supplementary Information. [file 41598_2022_7612_MOESM1_ESM.pdf]

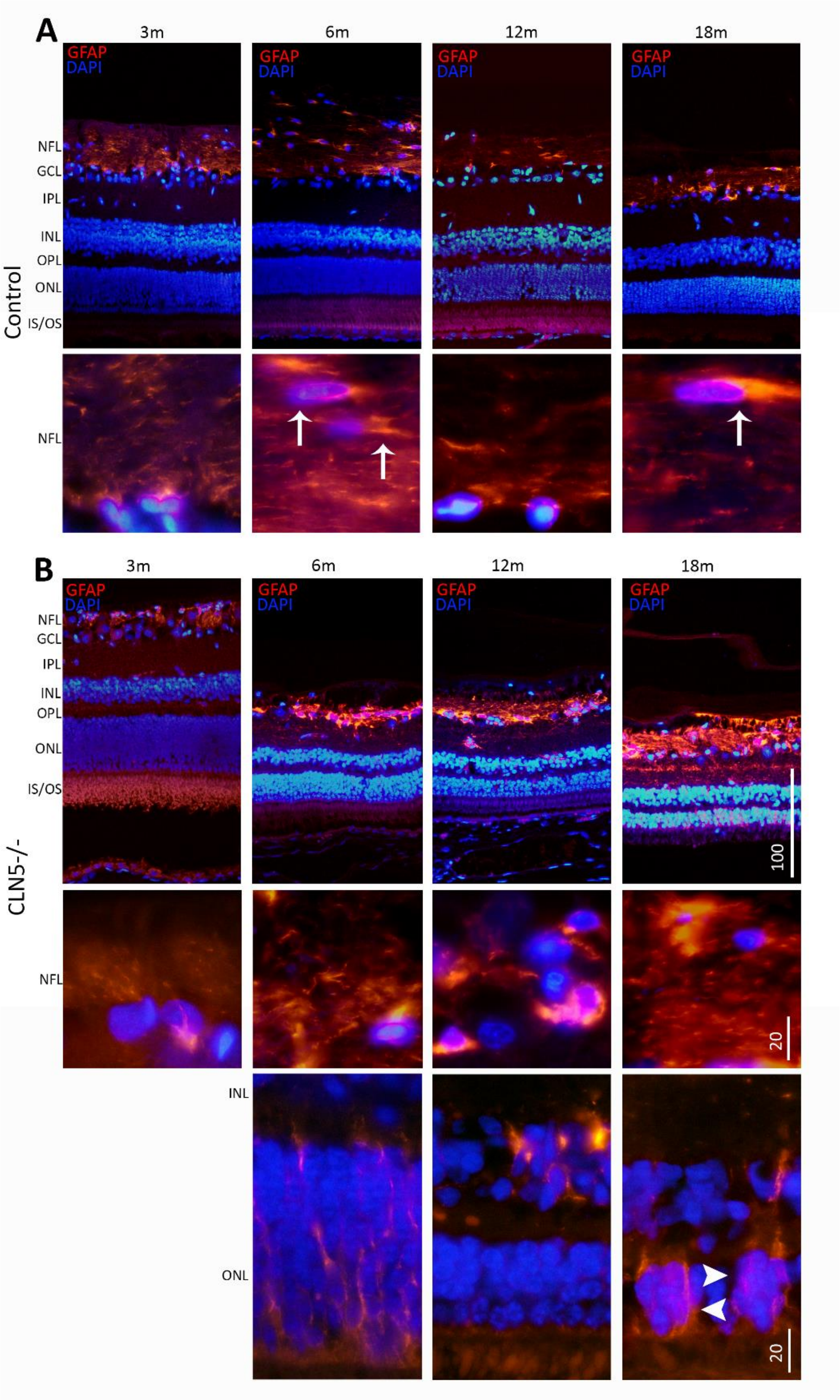

Figure S1. Astrocytic marker expression in the retina of healthy control and CLN5$^{-/-}$ sheep at different ages. A. Representative fluorescent images of control central retina at low (top panel) and high (bottom panel) magnification. **B.** Representative fluorescent images of CLN5$^{-/-}$ central retina at low (top panel) and high (bottom panel) magnification. GFAP; glial fibrillary acidic protein (red) with DAPI nuclei stain (blue). NFL; nerve fibre layer, GCL; ganglion cell layer, IPL; inner plexiform layer, INL; inner nuclear layer, OPL; outer plexiform layer, ONL; outer nuclear layer, IS/OS; inner and outer segments of photoreceptors, RPE; retinal pigment epithelium. Scale bar values are in µm.

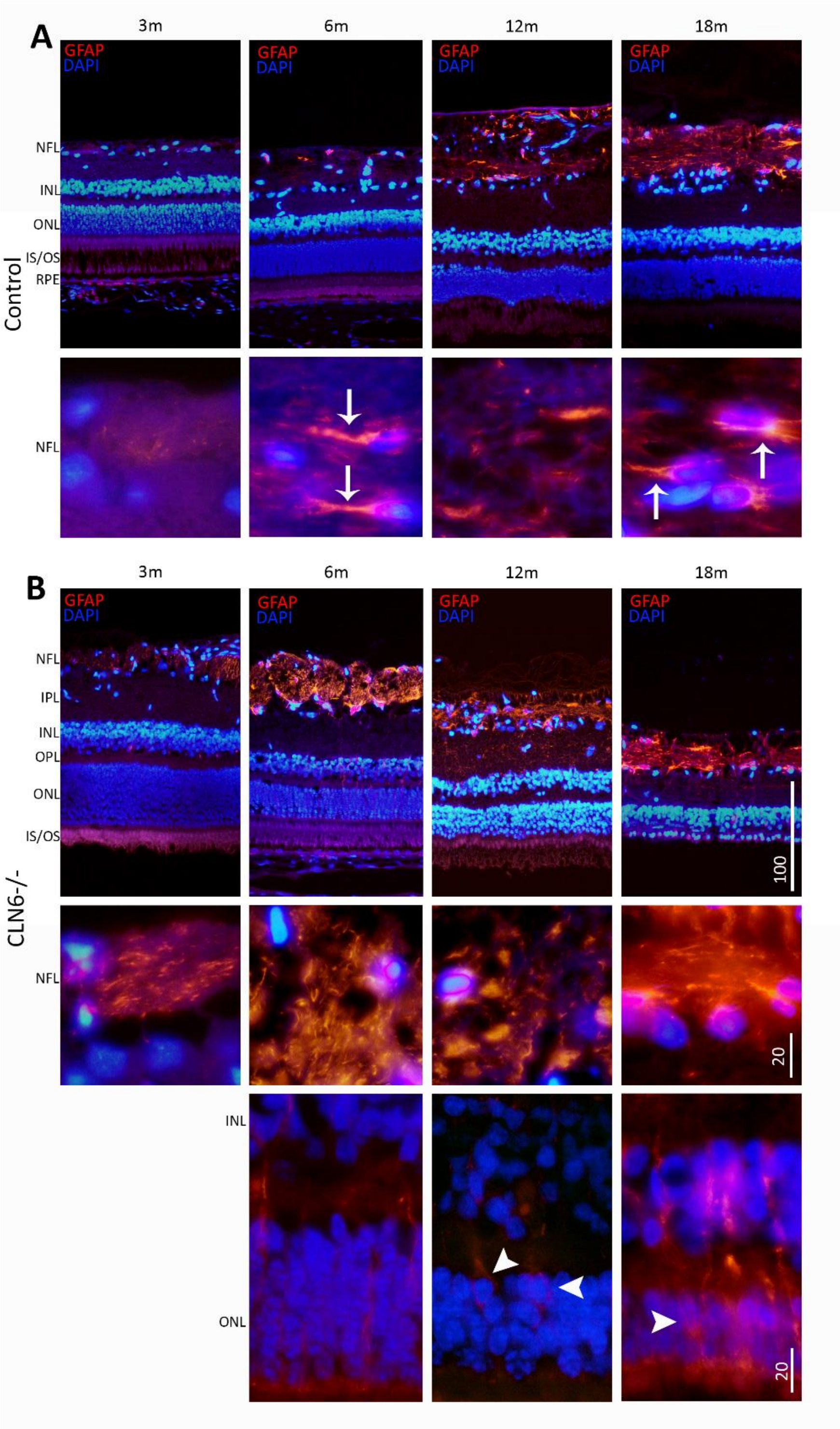

Figure S2. Astrocytic marker expression in the retina of healthy control and CLN6$^{-/-}$ sheep at different ages. A. Representative fluorescent images of control central retina at low (top panel) and high (bottom panel) magnification. **B.** Representative fluorescent images of CLN6$^{-/-}$ central retina at low (top panel) and high (bottom panel) magnification. GFAP; glial fibrillary acidic protein (red) with DAPI nuclei stain (blue). NFL; nerve fibre layer, GCL; ganglion cell layer, IPL; inner plexiform layer, INL; inner nuclear layer, OPL; outer plexiform layer, ONL; outer nuclear layer, IS/OS; inner and outer segments of photoreceptors, RPE; retinal pigment epithelium. Scale bar values are in µm.
